# Impact of Physical Fitness on Emergency Response: A Case Study of Factors That Influence Individual Responses to Emergencies among University Students

**DOI:** 10.3390/healthcare11142061

**Published:** 2023-07-19

**Authors:** Senka Bajić, Dragoljub Veljović, Borko Đ. Bulajić

**Affiliations:** 1 Faculty of Technical Sciences, University of Novi Sad, Trg Dositeja Obradovića 6, 21000 Novi Sad, Serbia; borkobulajic@uns.ac.rs; 2 Faculty of Sport and Physical Education, University of Novi Sad, Lovćenska 16, 21000 Novi Sad, Serbia; dragoljub.veljovic@gmail.com; 3 RISE Lab, 21000 Novi Sad, Serbia

**Keywords:** physical fitness, emergency response, disasters, preparedness, public health, general fitness assessment

## Abstract

(1) Background: The purpose of this study was to ascertain whether there is a direct correlation between the physical fitness of the general population, specifically students, and the response times to fire-emergency-related building evacuations and to identify which physical fitness factors more significantly influenced emergency movement times. (2) Methods: In this quantitative investigation, 21 students (both men and women of the same age) volunteered to participate. We first evaluated their physical fitness; then, we analyzed their reaction times and speed. (3) Results: The results of this study revealed a relationship between emergency response times and evaluations of muscular strength, muscular endurance, muscle power, cardiorespiratory fitness, and body composition. The physically active group demonstrated a stronger initial response (i.e., a shorter time to reach a safe location) to fictitious emergency scenarios. The reduction in the necessary response time did not, however, appear to be related to the degree of flexibility. (4) Conclusions: This study showed how physical fitness might alter initial emergency response times and lessen the effects of a disaster on the general population.

## 1. Introduction

One of the most important requirements during a disaster response is to move as rapidly as possible while taking the surroundings and the events into consideration; therefore, individual responses to an emergency may be of vital importance [1]. The first few seconds of an emergency are crucial with respect to taking actions that will improve an individual’s reaction to dangerous situations [2]. While it has been shown that emergency response teams can effectively conduct the minimization of the effects of disasters, people who find themselves in dangerous situations can still save their own lives by acting quickly before help arrives [3].

How individuals behave during the early phase of a fire is the most important survival factor in this regard [4], and the number of survivors is directly related to occupants’ reaction rates [5]. Recent research has revealed that when a building is fully inhabited, movement velocity is typically slow, particularly when people are descending stairs [6]. However, a fast response is essential for surviving fire situations. Rapid evacuation to a safe location is directly correlated with education, training, and other preparedness strategies [4]. The main focus of our investigation was to determine the physical factors that most greatly influence the rapid evacuation of individuals numbering among the general population and non-tactical personnel. 

The goal of this study was to identify the physical fitness factors that had the greatest impact on the participants’ emergency response times. Physical fitness assessments must be conducted in order to estimate an individual’s health status and physical preparedness. It is crucial to understand that physically demanding tasks necessitate increased levels of physical activity, which are directly associated with high levels of cardiovascular fitness as well as muscular strength and endurance [7,8]. The participants were divided into two groups based on their levels of physical activity: a group that was physically active and a group that was physically inactive. Thus, we were able to investigate whether people who are physically fitter complete evacuation routes more rapidly than those who are less fit. The uniqueness of this study primarily lies in its identification of the physical fitness characteristics that are related to movement speed during an emergency.

In the following sections, we will first present a review of the most important research relevant to this study, describe the method of analysis, and, finally, present the study itself, in which we will estimate the time required for each participant to reach a preset safe location during an evacuation in a scenario consisting of a staged fire in a building. Although the presented study refers to a staged fire scenario, we believe that our evacuation model can also be useful for the assessment of other similar simplified evacuation scenarios. 

## 2. Physical Activity vs. Response

Numerous studies have revealed that there is a strong correlation between actions taken during the emergency reaction phase and physical activity. In order to comprehend the relevance of physical fitness on individual emergency responses, it is essential to examine both general physical fitness metrics and initial reactions to an emergency. A more complex approach is used when describing physical performance in an emergency situation in relation to the occupational duties of emergency teams. To predict occupational performance, it is important to carry out both general fitness tests and job-specific tests [7]. All of those physically demanding job duties necessitate a high level of physical fitness [8]. Strength, endurance, and cardiorespiratory fitness represent the most important physical prerequisites for adequate occupational performance [9]. 

The main focus of this study was on the factors that affect how quickly non-emergency groups evacuate because it is believed that emergency teams benefit greatly from structured training programs with the primary goal of increasing and maintaining personal fitness [10]. 

The current study aims to ascertain how physical fitness may affect non-tactical personnel, namely, students between the ages of 20 and 30, who were randomly selected for the study. The emergency fire case scenario assumes that the selected students are attending lectures at a faculty building during a fire event. While the majority of studies that examine this topic concentrate on a behavioral model of emergency response teams and their physical abilities, this research highlights the research gaps in the areas of physical abilities, security, and the emergency responses of untrained citizens. Based on this knowledge, the goal of this study is to ascertain the extent to which the physical condition of students who had not participated in emergency response training or any physical activity prior to the emergency event affected their response times and amount of time required to reach a safe location. The effects of several fitness factors, such as body composition and levels of cardiorespiratory and muscular fitness (power, strength, endurance, and flexibility), were examined, and their impacts on response time in case of an emergency were compared. The participants were divided into two groups based on the data gathered from the exercise pre-participation health-screening questionnaire, and it was hypothesized that there would be substantial disparities in the answer times between the two groups.

## 3. Materials and Methods

### 3.1. Participants and the Protocol

The sample was made up of 21 students (both men and women of the same age) who volunteered to take part in the study. A pre-participation screening questionnaire was prepared by the researchers and used as a data collection form [11]. Following the *American College of Sports Medicine’s (ACSM) Exercise Preparticipation Health Screening Questionnaire for Exercise Professionals* [12], the participants were separated into two groups: a physically active group and a physically inactive group. This pre-participation screening form was created for use with the pre-participation screening algorithm provided by ACSM in their *Guidelines for Exercise Testing and Prescription*, 11th edition, 2022 [12,13]. 

Students from the Faculty of Technical Sciences at the University of Novi Sad were selected to take part in this study, none of whom were members of professionally trained emergency personnel. The key assumption was that each student’s degree of physical fitness would be closely related to how quickly they reacted in an emergency. Hence, we measured each participant’s height, weight, body composition parameters, level of cardiorespiratory fitness, level of muscular fitness (power, strength, endurance, and flexibility), and emergency response time. The first stage of the survey, during which we assessed physical fitness, lasted for four hours on a single day, while the second stage, during which we measured emergency reaction time and speed, was executed seven days later.

### 3.2. Risk-Factor Assessment and Body Composition Parameters

The pre-exercise evaluation included a measurement of solely resting blood pressure (BP). The *Procedures for Assessment of Resting Blood Pressure* recommended in the *ACSM Guidelines for Exercise Testing and Prescription* were used in this regard, as precise and proven methods for monitoring blood pressure are essential for accuracy. The results were successful, as all the subjects had normal blood pressure values according to the *Classification and management of blood pressure for adults*, allowing us to proceed with the testing. 

Low levels of physical activity can lead to an increase in weight, body fat, and associated health problems [14,15,16]. The dimensionless ratio of the circumferences of the waist and hips is known as the waist–hip ratio (WHR) or waist-to-hip ratio (WHR). The body mass index (BMI) is calculated by dividing the square of a subject’s height in meters by their body weight in kilograms. When BMI exceeds 25, a variety of obesity-related health issues can manifest, which worsen over time. A two-compartment model is used to express body composition as the proportion of fat and fat-free tissue with respect to total body mass [17]. Although body weight and waist and hip circumference (girth) can also be examined, the current study focused primarily on body composition and the skinfold procedure, which constitute a more accurate measurement of body fat. In this study, the percentage of body fat was calculated using a skinfold caliper. Measurements made using a caliper are thought to be more challenging but more accurate, and they yield a better estimate of body fat [17]. Body fat was measured on three skinfold sites in accordance with the *ACSM’s Guidelines for Exercise Testing and Prescription*—*Standardized Description of Skinfold Sites and Procedures* [17]. Using the three-site formula modified from Jackson and Pollock, pectoral, abdominal, and thigh skinfold sites were measured for the male participants, while for females, triceps, suprailiac, and thigh skinfold sites were measured [18].

### 3.3. Cardiorespiratory Fitness

Performance and functional statuses of the respiratory, cardiovascular, and skeletal muscle systems are intimately correlated with cardiorespiratory fitness. Maximal oxygen uptake (VO_2_max), that is, the highest quantity of oxygen the body can consume in a particular time period, is one of the established criteria for measuring cardiorespiratory fitness [19]. The choice of which submaximal or maximal exercise test to use for VO_2_max estimation mostly depends on the objectives of the test, the accessibility of the necessary tools, and the qualifications of the individuals conducting it. The Rockport One-Mile Fitness Walking Test, one of the most common and least strenuous tests for estimating cardiorespiratory fitness in a large population, was administered to subjects as part of this study. On a specific treadmill (*Life Fitness—Elevation series treadmill*), subjects were instructed to complete a mile (1.6 km) of walking as quickly as they could, and their heart rates were recorded immediately after completion of the test [17]. Heart rate was measured using heart rate monitors produced by reliable manufacturers (e.g., *The Polar heart rate monitor*). After the completion of the test, VO_2_max was estimated using a regression equation based on weight, age, sex, walking distance, and heart rate [20]:

VO_2_max = 132.853 + 6.3150 × Sx − 0.1692 × Bw − 0.3877 × Age − 3.2649 × Wt − 0.1565 × Hr (1)

where VO_2_max is calculated in mL/kg/min; Sx represents sex, with Sx = 0 for females and Sx = 1 for males; Bw is body weight in kg; Age is age in years; Wt is walking time in minutes; and Hr is heart rate in BPM (Beats per Minute). 

### 3.4. Muscular Strength

Muscular strength is the maximum force that a muscle or muscle group can produce at a particular velocity [19,21]. Strength assessments can be performed statically, where no obvious limb or muscular movement is required, or dynamically, where a load or body part is moved while a muscle changes length. Handgrip measurements have been proven to predict functional status despite static muscular strength assessments’ limitations in terms of representing overall muscular strength due to their focus on a single muscle group. For this test, peak force development is commonly described in terms of maximum voluntary contraction (MVC). Using a handgrip dynamometer (*Baseline 12-0241 Hand Dynamometer*), we tested static and isometric strength. Without holding their breath, the subjects were instructed to squeeze the dynamometer’s handgrip as tightly as they could (to avoid the Valsalva maneuver). After the test had been repeated twice with each hand, the value shown on the dynamometer was noted, and the score consisted of the highest value among all the readings [17].

### 3.5. Muscle Power

Power, i.e., the capacity or rate at which a subject can execute work, is a skill-related physical fitness factor [16]. In this study, the standing long jump, usually referred to as the broad jump, was measured to assess explosive leg power, which is frequently used to assess overall muscle power. Highly regarded sports associations (the *National football league, the National hockey league, etc.*) frequently use this test, which only needs a minimal amount of equipment and is straightforward to administer. The subjects were instructed to stand with their feet slightly apart behind a line drawn on the ground. Subjects were instructed to project their bodies forward by swinging both arms, with a two-foot takeoff and landing. Participants attempted to land on both feet without falling backward while jumping as far as they could. Following the completion of this maneuver, the distance between the take-off line and the subject’s heels’ closest point of contact was measured [22]. The longest distance jump was listed as the better of two tries.

### 3.6. Muscular Endurance

Muscular endurance is the capacity of a muscle group to carry out repetitive contractions for the amount of time necessary to induce muscular fatigue [17]. The maximum number of push-ups that could be performed without stopping was the simple field test used in this research to determine muscular endurance [21]. This method is used to assess upper-body muscular endurance. Male test participants were instructed to begin in the usual “down” posture (hands under the shoulders, back straight, head up, with the toes serving as the pivot point), while female test participants were instructed to begin in the modified “knee push-up” position. For this study, specialized equipment (*AssessPro Rep-Addition Push-Up Testers*) was employed to count the number of push-ups. The maximal number of push-ups performed without resting was noted as the final score. The test was stopped when the subjects could not maintain an appropriate technique and strained forcibly within two repetitions.

### 3.7. Flexibility

Flexibility is the capacity to move a joint across its full range of motion. The “Sit-and-reach” test, which measures lower back and hip joint flexibility, is one of the most frequently utilized tests [17]. Sit-and-reach tests may assess hamstring flexibility to a greater extent than lower back flexibility, but they can be used to examine health-related fitness until better criteria are available [23]. In this research, flexibility was measured using the “*Canadian Trunk Forward Flexion Test*”, which makes use of a sit-and-reach box that has been created specifically for the test. Following a brief warm-up and stretches, the subjects were instructed to sit with their feet flat on the flexometer without their shoes on for more accurate findings. Participants held that stance for two seconds as they slowly extended their hands forward as far as they could. From the best of two trials, the furthest point that the fingertips could reach was noted [17]. 

### 3.8. Emergency Evacuation Time 

In this study, the term “emergency response test” refers to each student’s performance in responding to an emergency scenario, specifically the measurement of the amount of time required to reach a safe location. The test was conducted in the classroom that the selected students frequented the most, which was located at the Department of Civil Engineering and Geodesy’s Laboratories at the Faculty of Technical Sciences in Novi Sad, Serbia. Based on how they responded to a fabricated emergency, the students’ performances and the actual times they needed to reach a safe area were evaluated. The main reason the test was not conducted in an actual emergency was due to several uncontrollable variables, including weather, a lack of trained personnel and emergency teams, the need for expensive equipment, and various psychological and sociological variables, all of which were avoided by employing a fictitious fire emergency. Moreover, because the external influences were avoided and controlled, we were able to establish a more accurate measurement of the response time and performance.

**Figure 1:**
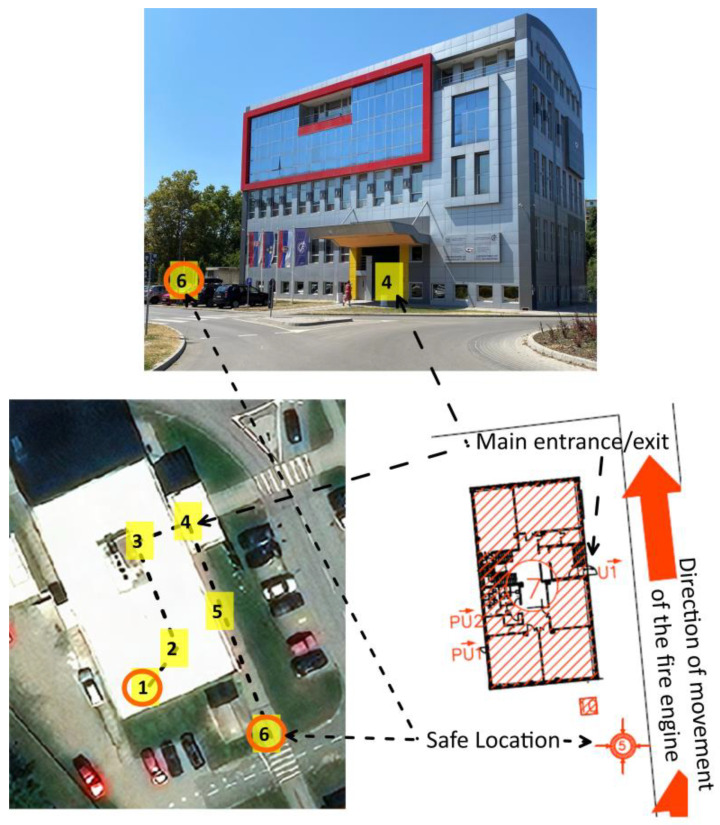
(**Top**)—view (from the north-east) of the selected building of the Faculty of Technical Sciences in Novi Sad; (**bottom left**)—aerial view of the building along with the designated scenario evacuation route shown in Figure 2; (**bottom right**)—partial presentation of the building’s fire protection plan.

The time it took a subject to leave the building and find a safe place was used as a measure of the initial emergency response. We established the length of time required for a subject to reach the safe area, which had been previously selected according to the building’s fire protection plan (Figure 1) [24]. 

**Figure 2:**
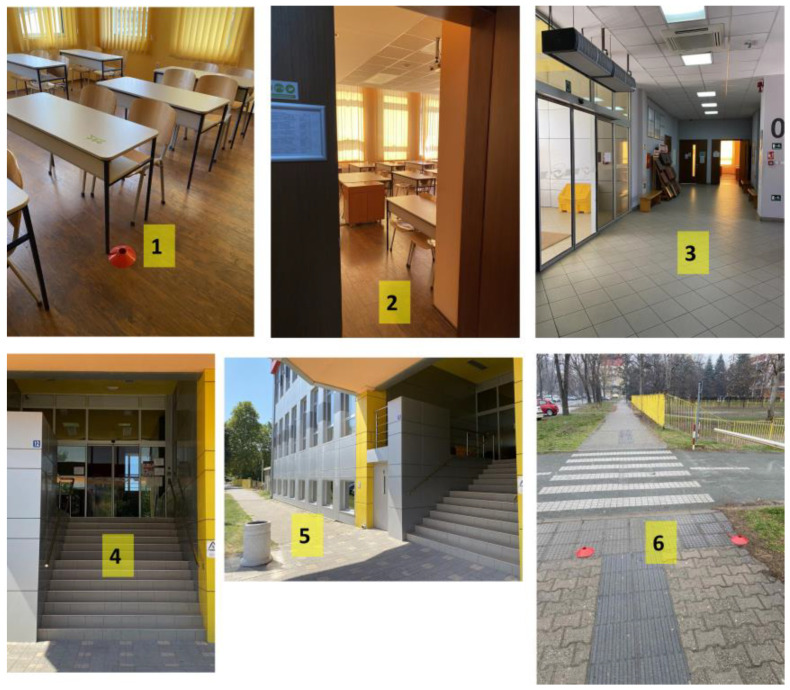
Evacuation route for the tested subjects according to the test scenario: (**1**) starting position in the selected classroom, (**2**) entrance to the classroom, (**3**) the hall between the classroom and the main entrance, (**4**) the main entrance to the building and the 13 stairs to the exterior of the building, (**5**) path from the entrance to the safe location, and (**6**) the safe location.

Individual tasks were completed by each participant in order to produce results that were more accurate for each person. Various researchers have demonstrated that the initial response time measured from the moment the emergency call or fire alarm is detected can be used as a direct indicator of how well the emergency teams function and how well the effects of disasters are mitigated [3,25]. Thus, in this research, we followed the same procedure for the general population. The time that passes between when an incident is reported (via a fire alarm) and when a subject arrives at a safe area was used as a performance indicator. The sound of the fire alarm was the indicator for the participant to begin evacuating. Before the test, the subjects, each of whom were students, were acquainted with the procedure and the safest route (all participants took the same evacuation route) that could take them to the safe location outside of the building. Subjects were required to sit in a chair in a classroom on the elevated ground floor. According to the fire protection plan, students were required to pass the main entrance door, descend 13 stairs to the exterior of the building, and then run or walk the remaining distance from the starting point to the safe location, which was situated 30 m away from the entrance (Figure 2). The total distance of the entire evacuation route was 55 m. The chair case in the center of the classroom served as the starting point for all the subjects, and it was marked with markers. The finishing position was in the safe location, and it was similarly marked with two clearly visible markings. The subjects were advised to move to the finishing point upon hearing the fire alarm, which signaled the beginning of the test. The trained staff stationed at the finishing point measured the response times. Electronic timing gates were not available, so the measurement of response time was performed using a handheld stopwatch. The time was measured in seconds, as even a few seconds in an emergency can be crucial [2]. The distance from the starting position to the finishing point in the safe location was measured prior to the test.

### 3.9. Statistical Analysis

The *Statistical Package for Social Sciences* (SPSS, Inc.) version 16.0 was utilized to perform data analysis. The two-tailed Student’s t-test was used to assess whether there was a significant difference between the means of the two groups and the test hypotheses regarding the mean of a small sample drawn from a normally distributed population (normal/Gaussian distribution). The approach outlined herein examined disparities between physically active students and physically inactive students. The variables of interest were body composition (percentage of body fat), muscular strength (handgrip), muscle power (standing long jump), muscular endurance (push-ups), flexibility, cardiorespiratory fitness, and the outcome of the emergency reaction test, in this case, a 55 m movement test. According to the *American College of Sports Medicine* (ACSM) for Exercise Professionals [12], the group was divided into two smaller groups: physically active students (10 subjects) and physically inactive students (11 subjects). The equality of variances for a variable calculated for two groups was evaluated using Levene’s Test for Equality of Variances. The *p*-value significance threshold was set at *p* ≤ 0.05.

## 4. Results

The descriptive data for all 21 students (male and female), across all measures, are shown in Table 1, with no data being excluded from the analysis. Table 2 compares emergency reaction times for physically active and physically inactive students with regard to a variety of physical variables.

The basic assumption of this study, namely, physically active students would have shorter emergency response times than physically inactive students, is the main justification for the use of the two-tailed test. Additionally, the research’s primary focus was on the physical factors that have the greatest impact on emergency response rather than whether or not people who are physically active will respond to emergencies more quickly. Among all the physical variables that were tested, the highest degrees of comparative significance were found for body fat percentage, muscular strength, muscle power, muscular endurance, cardiorespiratory fitness, and emergency response time. These results suggest that the most crucial factors in determining initial emergency response times and performance for untrained young adults are body composition, handgrip strength, standing long jump performance, push-up performance, and performance regarding the Rockport One-Mile Fitness Walking Test. However, when comparing the emergency response times of physically active and sedentary (physically inactive) students, factors like body mass index and degree of flexibility did not show any appreciable variations.

Students who were physically active completed a 55 m movement considerably (*p* ≤ 0.05) faster and with a shorter emergency response time; in this case, the *p*-value was 0.001. Second, physically active students had a lower body fat percentage, with a *p*-value of 0.003. Third, physically active students performed better on the test of muscular strength, specifically a handgrip test presenting a total score for both hands, and the corresponding *p*-value was equal to 0.002. Fourth, physically active students performed better on the standing broad jump test, which was associated with a *p*-value of less than 0.001. Finally, with a *p*-value of less than 0.001, physically active students performed better on the muscular endurance test (push-ups) than physically inactive students. Furthermore, with a *p*-value of 0.027, the cardiorespiratory fitness test showed that physically active students also performed better. Physical parameters such as flexibility, however, did not differ significantly between physically active students and those who were not. The main study hypothesis was confirmed by the data, which show that the emergency reaction times are quicker for the physically active students.

## 5. Discussion

The major goal of this study was to identify the precise physical fitness parameters that could have a direct impact on evacuation time in accordance with the premise that individuals who are more physically fit finish evacuation routes more quickly than those who are less physically fit. Despite the fact that evacuation simulation tools have long allowed users to assume various movement speeds of evacuees, the uniqueness of the present findings primarily lies in its identification of the physical fitness characteristics that are related to movement speed. Although physical activity is still not formally recognized as a disaster preparedness activity, according to the results presented herein, untrained employees who are engaged in physical activity could perform better in the disaster evacuation process. Recent research has indicated that even four-week physical fitness programs could enhance measures of physical fitness and performance in terms of simulating occupationally specific tasks among tactical athletes, leading researchers to draw the conclusion that the general population would also gain from physical training programs during the emergency response phase [26]. Further in-depth analysis of the uncontrolled environment of evacuation drills would likely produce more accurate findings. 

Some institutions, including Boston University, recommend a speedy but peaceful evacuation: “Evacuate calmly and quickly whenever a fire alarm or carbon monoxide alarm sounds” [27]. Slower evacuation times might indicate familiarity with fire evacuation procedures, experience, and a deliberate and calm movement to the outside evacuation point, which is what such institutions would normally advise. In actual evacuation events, people tend to desire to escape a building as soon as possible, presenting varied and strained body sensations while responding to a threat, so the expectation of a quiet evacuation may change. Future research should focus on how physical activity may affect a more peaceful evacuation process in terms of psychology.

After the fire alarm sounded, the subjects were instructed to head as quickly as they could to the finish line, simulating the conduct of people actually threatened by fire. The impact of this research’s findings could offer not only an overview of the primary physical factors that might affect disaster responses, along with those that should be given special attention in designing training programs for the general population, but also the realistic behavior of untrained participants. 

Although we acknowledge that the key result of this study, i.e., it takes longer for less physically fit people to evacuate using a predetermined route, may not be a novel one, a new study that shows the significance of physical activity, particularly physical fitness factors, with respect to disaster responses will affect how people perceive it as an essential component of emergency preparedness. Physical activities, which have been shown to be a very important factor in safer evacuation, may also, as previously mentioned, boost self-confidence, lead to calmer behavior, and decrease panic states.

The data used in this study offered a unique opportunity to examine and contrast two groups of students who were only separated by their levels of physical activity. The results of this study clearly suggest that some physical aspects of emergency reaction time—which has been shown to be shorter among physically active students—have a stronger impact than others and should be taken into consideration when developing a particular physical activity program.

Muscle power and endurance are the factors that revealed the most significant differences between the two groups in addition to a quicker emergency reaction among physically active students. The muscle power test is one of the most often used tests to ascertain the physical features of emergency personnel, and it is also one of the most important performance indicators [28]. Because disaster-related situations necessitate high-intensity movements, power appears to be essential for emergency performance [29]. Peak power can be tested using a variety of techniques, and future research may concentrate on the vertical jump or the medicine ball throw test, which can also demonstrate whether muscle power directly affects the emergency reaction.

Muscular endurance can also be tested in a variety of ways. Push-ups are a useful performance indicator since elite tactical units have shown greater muscular endurance when performing push-ups compared to the general population [28]. The results of this study can be considered to prove the fact that endurance training increases physical capacity and performance for a variety of emergency occupational tasks [30]. 

The handgrip test, which served as an indicator of muscular strength in this study, distinguished between physically active students and those who were not. Strength training is one of the most important components of the physical ability test that candidates for fire departments must pass. Numerous studies have demonstrated how the level of physical fitness affects the field duty of emergency workers, particularly firefighters, in many disaster events [14,27]. Handgrip strength is also favorably correlated with successful aging among older persons, meaning that maintaining a high degree of muscular strength in the general population may be helpful in dealing with the challenges of aging [31]. Future research can focus on enhancing muscular strength tests by including one-repetition maximum tests (1RM tests), which can increase the precision and accuracy of results.

Even though there have been studies showing that cardiorespiratory fitness is statistically significant in terms of predicting emergency reactions among trained emergency workers [3], it was anticipated that cardiorespiratory fitness would also have a direct impact on the initial emergency response among members of the untrained population. According to the results of the current study, students who were more physically active and had higher levels of average cardiorespiratory fitness were able to perform better than students who had lower average cardiorespiratory fitness as demonstrated by their shorter evacuation times. Future studies might concentrate on VO_2_max estimation using a maximal cardiorespiratory fitness test, which could yield more accurate results.

Many of the physical variables examined in this study are influenced by major factors related to body composition. This study found that students with quicker emergency response times had lower body fat percentages. As the most-used anthropometric approach for estimating body composition is skinfold measurement, the results of this study serve as a realistic indicator [32]. Additionally, lean mass is thought to be related to gains in strength, which suggests that increased strength is directly related to body composition [14]. This study found that higher levels of body fat had an impact on emergency response times, suggesting that students who are inactive or have low levels of physical activity can increase their ability to react to a disaster by engaging in prescribed physical exercises.

### Limitations

The participants of this research study do not constitute a very homogenous group in terms of age and fitness and thus may not accurately represent the general population of evacuees in the majority of buildings within one settlement, and this is one of the study’s limitations. On the other hand, the participants might represent the broader populace with respect to various university campuses and structures. The senior population of office and industry workers, who may represent a wider age profile and have more health issues, could be included in a future study, and their inclusion could have an impact on evacuation procedures. Although the study’s participants represented a small sample, we believe a credible analysis was conducted, simulating a real scenario in which many people could truly be accommodated in only a single ordinary university classroom.

Some of the physical variables examined in our study, such as muscle power and body composition, may be more closely related to an individual’s response to an emergency (movement time). Considering that future studies may examine the movement times of participants who must evacuate from the higher floors of buildings, where their movement times could be longer and, consequently, directly affected by cardiorespiratory fitness, it is important to address the fact that the emergency response time of the participants in this study was only 10–20 s and thus would not entirely be affected by a person’s VO_2_max. Future studies might also concentrate to a greater extent on how quickly people react in an emergency rather than just how long it takes them to proceed to a safe location and analyze how emergency reaction speed correlates with various aspects of physical fitness.

According to the literature, the choice of path depends on how familiar occupants are with a building. The participants in this study were very familiar with the building used for the test and, therefore, experienced little difficulty in orienting themselves within the building. This can be perceived as a limitation of this study because evacuation performance is determined by perception and prior knowledge of a building [4]. Furthermore, if the test was conducted in an unfamiliar building, the movement time would probably be longer.

The majority of the physically active participants engaged in fitness activities (gym) and recreational team sports like soccer and football. Future studies might focus on examining the same types of physical activity and a particular training regimen so that the precise impact of physical activity on a person’s emergency response can be determined.

The focus of this research was on assessing general fitness, and some of the physical fitness characteristics examined, such as muscular strength (hand grip) and muscular endurance (push-ups), did not directly correspond with the individual emergency movement times (55 m performances). This could be our study’s major weakness because other studies have found that the results of individual physical assessment tests have little predictive value with respect to actual emergency performance. Contrarily, fitness component tests have construct validity and are conducted according to standards that are widely acknowledged by the scientific community [18]. This research could potentially serve as a guide for future investigations into the development of specialized protocols that could investigate the significance of a population’s individual emergency reaction times.

## 6. Conclusions

Supposing performance ability is a crucial component for emergency teams, this study examined whether participants who were physically active and responded to a simulated emergency more quickly also presented higher values for various fitness-related metrics. The findings of this study imply that measurements of physically active individuals’ muscular strength, endurance, and power; cardiorespiratory fitness; and body composition may have an impact on their emergency response times.

Although there is no requirement for physical activity imposed on the students at the Faculty of Technical Sciences in Novi Sad, given the importance of the shown connection between disaster response and physical activity, it could be suggested that at least the physically inactive participants in this study and their colleagues should become more active in the future. Future research should concentrate on the connection between emergency drills and physical fitness, which can be seen as a key component of training and better preparedness for any disaster. Some studies have shown that there is a direct correlation between increased preparedness and the decreased vulnerability of the at-risk population, demonstrating the benefits of disaster drills and training [33]. Besides participating in disaster preparedness training, which will improve their performance in emergencies, people should be physically active in order to maintain their body and mental health and be ready to respond appropriately in an emergency.

## Figures and Tables

**Table 1:** Descriptive statistics for all physical fitness test variables and movement times for two groups.

Variable	Group	Mean	Std. Deviation	Std. Error Mean
Body mass index (kg/m^2^)	Physically active	25.0400	3.61669	1.14370
	Physically inactive	25.0182	5.62207	1.69512
Body fat (%)	Physically active	13.3310	5.80855	1.83682
	Physically inactive	23.8091	7.95807	2.39945
Hand grip (total) (kg)	Physically active	100.5000	28.08024	8.87975
	Physically inactive	64.6364	18.38082	5.54203
Standing broad jump (cm)	Physically active	207.2000	23.36094	7.38738
	Physically inactive	147.4545	31.87590	9.61094
Push-ups (repetitions)	Physically active	23.9000	7.92254	2.50533
	Physically inactive	10.2727	6.23042	1.87854
Flexibility (cm)	Physically active	32.0500	8.85516	2.80025
	Physically inactive	29.0455	8.68463	2.61851
VO_2_max (mL/kg/min)	Physically active	44.5500	7.02745	2.22227
	Physically inactive	36.2364	8.65139	2.60849
Movement time 55 m (s)	Physically active	13.0640	0.98135	0.31033
	Physically inactive	16.0336	2.09683	0.63222

**Table 2:** Comparative statistics for all measures: results of the two-tailed Student’s t-test for all evaluated physical fitness variables and movement times for the two groups.

Variable	Levene’s Test for Equality of Variances		*t*-Test for Equality of Means						
	F	Sig.	t	df	Sig. (2-Tailed)	Mean Difference	Std. Error Difference	95% Confidence Interval of Difference F	
								Lower	Upper
Body mass index (kg/m^2^)	0.612	0.444	0.010	19	0.992	0.02182	2.08776	−4.34792	4.39156
Body fat (%)	1.353	0.259	−3.415	19	0.003	−10.47809	3.06830	−16.90011	−4.05607
Hand grip (total) (kg)	2.120	0.162	3.496	19	0.002	35.86364	10.25922	14.39084	57.33644
Standing broad jump (cm)	0.695	0.415	4.855	19	≤0.001	59.74545	12.30628	33.98812	85.50279
Push-ups (repetitions)	0.716	0.408	4.404	19	≤0.001	13.62727	3.09458	7.15024	20.10430
Flexibility (cm)	0.367	0.552	0.784	19	0.442	3.00455	3.83006	−5.01187	11.02096
VO_2_max (mL/kg/min)	0.243	0.628	2.401	19	0.027	8.31364	3.46214	1.06730	15.55997
Movement time 55 m (s)	3.984	0.060	−4.083	19	0.001	−2.96964	0.72723	−4.49174	−1.44753

## Data Availability

Data are contained within the article.
